# Understanding 3D structural complexity of individual Scots pine trees with different management history

**DOI:** 10.1002/ece3.7216

**Published:** 2021-01-31

**Authors:** Ninni Saarinen, Kim Calders, Ville Kankare, Tuomas Yrttimaa, Samuli Junttila, Ville Luoma, Saija Huuskonen, Jari Hynynen, Hans Verbeeck

**Affiliations:** ^1^ Department of Forest Sciences University of Helsinki Helsinki Finland; ^2^ School of Forest Sciences University of Eastern Finland Joensuu Finland; ^3^ CAVElab ‐ Computational & Applied Vegetation Ecology Department of Environment Faculty of Bioscience Engineering Ghent University Gent Belgium; ^4^ Natural Resources Institute Finland Helsinki Finland

**Keywords:** box dimension, forest ecology, ground‐based LiDAR, growth and yield, silviculture, terrestrial laser scanning, tree structure

## Abstract

Tree functional traits together with processes such as forest regeneration, growth, and mortality affect forest and tree structure. Forest management inherently impacts these processes. Moreover, forest structure, biodiversity, resilience, and carbon uptake can be sustained and enhanced with forest management activities. To assess structural complexity of individual trees, comprehensive and quantitative measures are needed, and they are often lacking for current forest management practices. Here, we utilized 3D information from individual Scots pine (*Pinus sylvestris* L.) trees obtained with terrestrial laser scanning to, first, assess effects of forest management on structural complexity of individual trees and, second, understand relationship between several tree attributes and structural complexity. We studied structural complexity of individual trees represented by a single scale‐independent metric called “box dimension.” This study aimed at identifying drivers affecting structural complexity of individual Scots pine trees in boreal forest conditions. The results showed that thinning increased structural complexity of individual Scots pine trees. Furthermore, we found a relationship between structural complexity and stem and crown size and shape as well as tree growth. Thus, it can be concluded that forest management affected structural complexity of individual Scots pine trees in managed boreal forests, and stem, crown, and growth attributes were identified as drivers of it.

## Graphical abstract

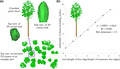

## 1 INTRODUCTION

Forests are the largest terrestrial ecosystem covering one third of the earth's surface area (Roxburgh and Noble, 2013), and they provide a range of services such as carbon uptake (Hardiman et al., 2011), productivity (Puettmann et al., 2015), biodiversity (Fedrowitz et al., 2014), and resilience (Messier et al., 2013). Processes of growth and regeneration are closely related to these services also linking them with forest structure (von Gadow et al., 2012). The current forest structure is a result of tree and stand dynamics affected by the availability of resources such as light, nutrients, and water, and by the competition of these resources. Both biotic (e.g., insects, pathogens) and abiotic (e.g., fire, wind, snow) disturbances, and forest management and changing climate alter relationships between trees through changes in these growing conditions (i.e., availability of light, nutrients, and water) and therefore stand dynamics and forest structure.

Trees are interacting with each other and that affects their functioning and structure. Tomlinson (1983) has pointed out that the development of trees and their structure can therefore enhance our understanding about forest structure. Thus, investigations on individual trees are important. Trees occupy three‐dimensional space, and tree architecture can be characterized based on growth dynamics and branching patterns (Tomlinson, 1983). Tree structure, on the other hand, can be characterized by using morphological measures such as crown dimension (e.g., volume, surface area) and stem attributes (e.g., diameter at breast height (DBH), height, height of crown base) (Pretzsch, 2014). The availability of 3D point clouds from terrestrial laser scanning (TLS) has provided an effective means for such measurements allowing TLS to be utilized in generating stem and crown attributes (Bayer et al., 2013; Calders et al., 2013, 2018; Georgi et al., 2018; Juchheim et al., 2017; Liang et al., 2012; Metz et al., 2013; Saarinen et al., 2017, 2020; Seidel et al., 2011). However, objective and quantitative measures for structural complexity of individual trees are needed to better understand relationship between forest structural diversity and ecosystem services such as biodiversity, productivity, and carbon uptake (Hardiman et al., 2011; Messier et al., 2013; Puettmann et al., 2015; Zenner, 2015).

**Figure 1:**
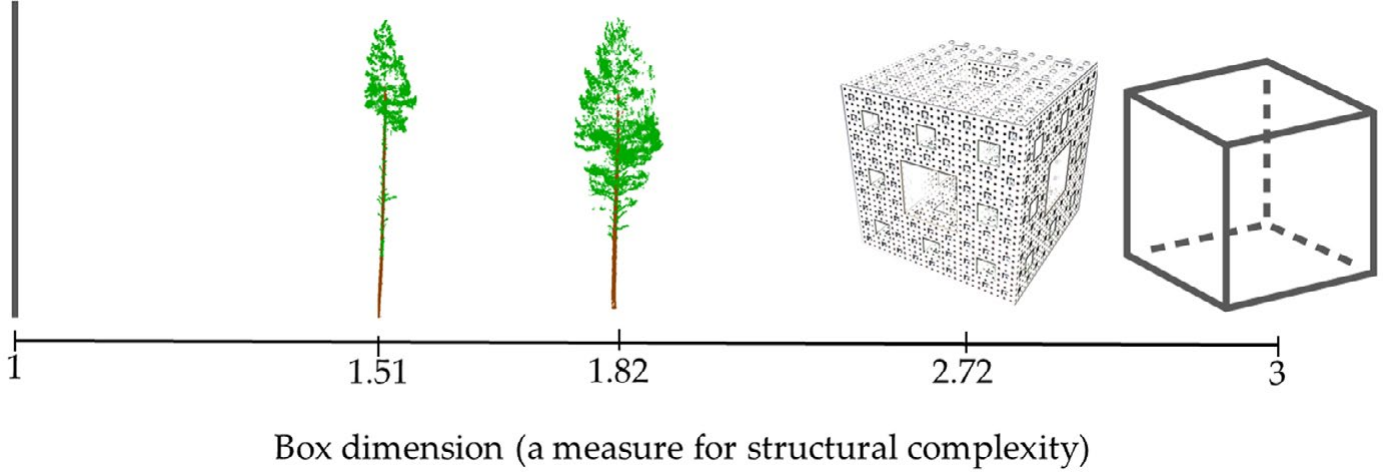
Examples of objects with box dimension ranging from one (cylindrical pole) to three (solid cube) in between two real‐life trees and a Menger sponge (box dimension = 2.72). Modified after Figure 1 in Seidel, Annighöfer, et al. (2019)

Fractal analysis (Mandelbrot, 1977; Shenker, 1994) can provide an approximation of natural forms, and TLS has opened possibilities for applying fractal analysis for characterizing structural complexity of individual trees (Calders et al., 2020). Seidel (2018) presented an approach where fractal analysis of Minkowski–Bouligand dimension (or box‐counting dimension, that is, changes in number of boxes required covering an object when the boxes are made more defining) was applied in characterizing structural complexity of individual trees. Even before TLS existed, the so‐called box dimension was used to characterize spatial patterns of foliage distribution with plastic flaps of different sizes to measure the presence of leaves (Osawa & Kurachi, 2004). Seidel (2018) used boxes (or voxels) of different sizes to enclose all 3D points from individual trees obtained with TLS, whereas Osawa and Kurachi (2004) used cylinders for estimating box dimension. Regardless of the geometric primitive, the box dimension is determined as a relationship between the number of primitives of varying size needed to enclose all 3D points of a tree and the inverse of the primitive size. The box dimension is scale‐independent and can theoretically vary between one and three, one being a cylindrical, pole‐like object and three corresponding solid objects such as cubes (Figure 1). Seidel, Annighöfer, et al. (2019) assumed that maximum box dimension value for trees would be 2.72 that corresponds to the fractal object of a Menger sponge, which has infinite surface area with zero volume (Mandelbrot, 1977; Pickover, 2009).

Box dimension is a relatively new measure for assessing structural complexity of trees and forests in relation to TLS. Seidel, Ehbrecht, et al. (2019) studied the relationship between structural complexity (i.e., box dimension) and horizontal and vertical architectural characteristics (i.e., tree height and volume, crown radius and surface area, branching angles) of deciduous trees (*Fagus sylvatica, Fraxinus excelsior, Acer pseudoplatanus, Carpinus betulus*) of varying size. They concluded that structural complexity was related to crown radius and surface area of deciduous trees. Dorji et al. (2019) studied how competition affects structural complexity of European beech (*Fagus sylvatica* L.) trees and concluded that their crowns were influenced by competition, as measured through box dimension. Seidel, Annighöfer, et al. (2019), on the other hand, reported decreasing structural complexity when competition (i.e., light availability) increased for deciduous trees. Previous work (Seidel, 2018, Seidel, Annighöfer, et al., 2019); Seidel, Ehbrecht, et al., 2019) demonstrated the potential of box dimension as a meaningful measure for structural complexity of individual trees. However, how this measure can be used to quantify forest structure of conifers and how it can expand our understanding about effects of anthropogenic activities (e.g., forest management) on tree structure are largely unexplored.

Although forest management affects growing conditions of trees, and their size and shape (Mäkinen & Isomäki, 2004; Saarinen et al., 2020), it is unclear how forest management affects structural complexity of conifers. This study aimed at identifying relationships between a variety of attributes (e.g., characterizing stem, crown, and competition) and structural complexity of individual Scots pine (*Pinus sylvestris* L.) trees in even‐aged and single‐layered managed boreal forest conditions. We aimed to understand how structural complexity is driven by forest management and underlying structural attributes. We hypothesize that thinning intensity affects structural complexity of Scots pine trees (H1). It is also hypothesized that horizontal and vertical measures (e.g., stem and crown dimensions) are related to structural complexity of Scots pine trees (H2). Finally, we hypothesize (H3) that there is a relationship between structural complexity and (a) crown dimensions, (b) architectural benefit‐to‐cost ratio (i.e., surface‐to‐volume ratio), (c) tree growth (i.e., DBH, height, volume, and Δheight/DBH), and (d) the availability of light (i.e., competition). In other words, structural complexity of individual Scots pine trees can be explained by these attributes.

## 2 MATERIALS AND METHODS

### 2.1 Study site and data acquisition

**Figure 2:**
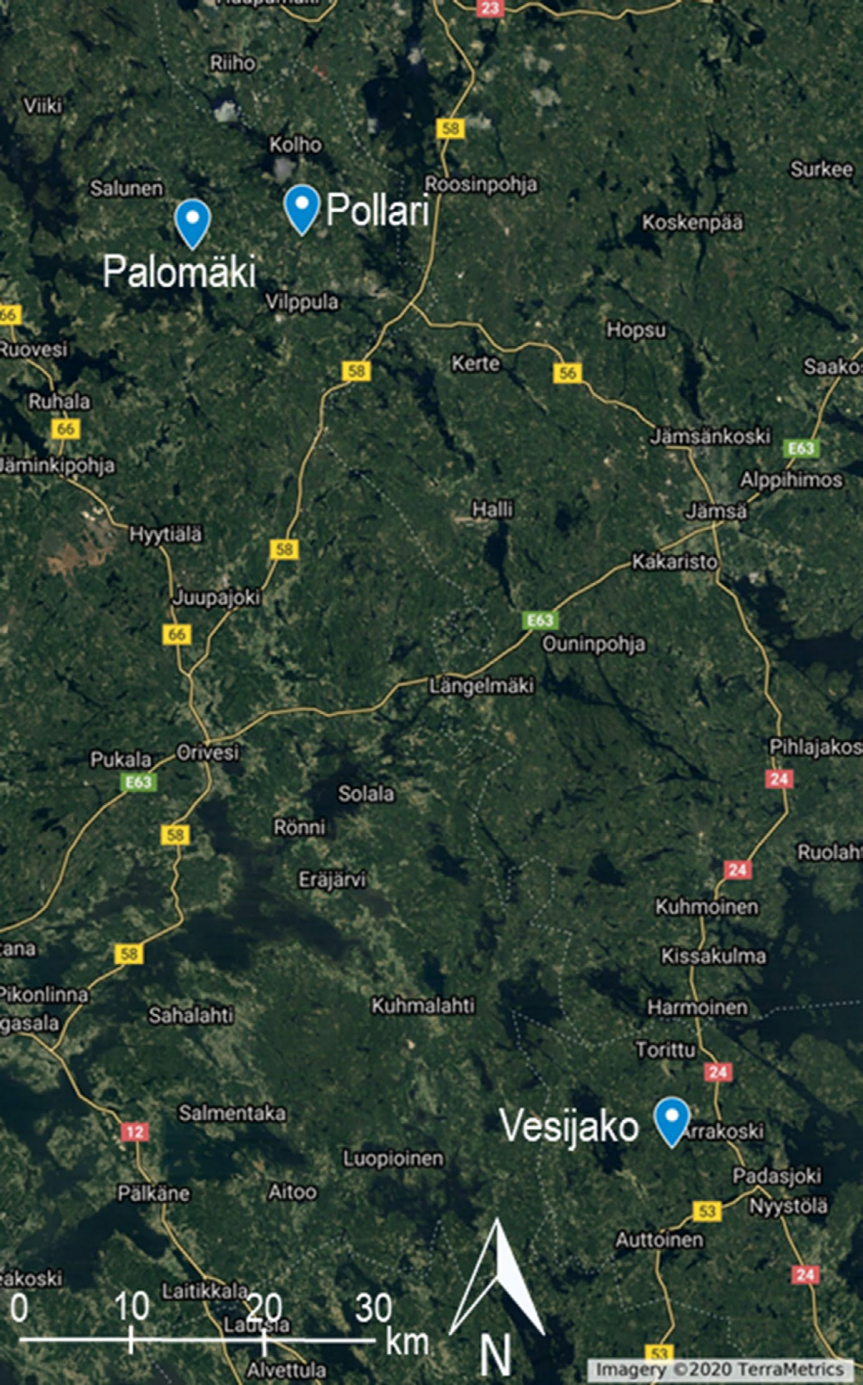
Location of the three study sites namely Palomäki, Pollari, and Vesijako and vegetation zones in Finland (left) and study sites on top of satellite imagery © 2020 TerraMetrics

The study area consists of three study sites dominated by Scots pine (Palomäki, Pollari, and Vesijako) (Figure 2), established and maintained by Natural Resources Institute Finland (Luke). All study sites are located in southern boreal forest zone and characterized as mesic heath forest (i.e., Myrtillus forest site type according to Cajander (1913)). Nine rectangular sample plots (size from 900 and 1,200 m^2^) were placed at each study site resulting in a total of 27 sample plots. At the time of the establishment (Palomäki in 2005, Pollari and Vesijako in 2006), the stand age was 50, 45, and 59 years for Palomäki, Pollari, and Vesijako, respectively. The first thinning (removal ~30% of stems) had been carried out for all study sites in the early 1990s.

The experimental design of the study sites includes two varying levels of thinning intensity (i.e., moderate and intensive), and one plot at each study site remained as a control plot where no thinning has been carried out since the establishment of the sites. The remaining relative stand basal area after moderate thinning was ~68% of the stocking before thinning, and intensive thinning reduced the stocking levels down to 34%. Suppressed and codominant, and unsound and damaged (e.g., crooked, forked) trees were removed (i.e., thinning from below) in both thinning intensities. Other thinning treatments were also carried out, but here, we concentrated on three plots with moderate, three plots with intensive, and three plots with no treatment since establishment.

The plots were measured and thinning treatments performed at the time of the establishment (in 2005 at Palomäki and in 2006 at Pollari and Vesijako). The most recent field measurements were carried out in October 2018 and in April 2019. Tree species, crown layer, DBH from two perpendicular directions, and health status were recorded from each tree within a plot (i.e., tally trees). At the time of the latest measurements, the proportion of Norway spruce and deciduous trees (i.e., *Betula* sp and *Alnus* sp) from the total stem volume of all trees within the nine sample plots was 3.02% and 0.07%, respectively. Approximately half of the trees (*n* = 318) were selected as sample trees from which tree height, live crown base height (i.e., the height of the lowest live branch), and height of the lowest dead branch were also measured. Height of the tally trees was estimated using an allometric model calibrated for each sample plot with the information from the sample trees. Stem volume of individual trees was produced by using the existing nationwide, species‐specific volume equations with DBH and height as predictors (Laasasenaho, 1982) to obtain corresponding information from different field measurements. Plot‐level attributes before and after thinning treatments are presented in Table 1, and the development of tree‐level attributes for each thinning treatment can be found in Table 2.

**Table 1:** Mean stand characteristics by treatments before and after thinning and thinning removal

	Before thinning (2005–2006)			After thinning (2005–2006)		
	Moderate	Intensive	No treatment	Moderate	Intensive	No treatment
*N*/ha	1,269	1,244	1,337	716	289	1,337
*G* (m^2^/ha)	26.5	26.5	27.7	18.1	8.7	27.7
*D*_w_ (cm)	17.5	18.0	17.8	18.7	20.4	17.8
*H*_w_ (m)	16.1	16.3	16.1	16.5	16.9	16.1
*H*_100_ (m)	17.3	17.7	17.5	17.3	17.5	17.5
*V* (m^3^/ha)	213.4	215.7	224.0	148.3	72.9	224.0

Note

*N* = stem number per hectare, G = basal area, *D*_w_ = mean diameter weighted by basal area, *H*_w_ = mean height weighted by basal area, *H*_100_ = dominant height, and *V* = volume.

**Table 2:** Mean tree‐level attributes with its standard deviation (with ±) and range in square brackets for each treatment at the year of the establishment (first measurement) and the last measurement

	First measurement (2005–2006)			Last measurement (2018–2019)		
	Moderate	Intensive	No treatment	Moderate	Intensive	No treatment
DBH (cm)	17.6 ± 3.3 [10.3; 28.2]	19.3 ± 3.4 [11.7; 29.8]	15.4 ± 4.6 [5.8; 30.7]	22.2 ± 3.7 [13.4; 35.3]	26.4 ± 3.9 [17.9; 36.4]	18.7 ± 5.0 [6.5; 34.4]
Height (m)	15.9 ± 1.9 [11.9; 19.9]	16.5 ± 1.8 [13.0; 20.5]	14.7 ± 2.6 [9.0.7; 23.0]	21.2 ± 2.1 [14.7; 25.9]	21.2 ± 1.7 [18.2; 25.3]	20.0 ± 3.0 [13.2; 30.3]
Volume (dm^3^)	202.7 ± 89.3 [50.9; 549.4]	249.1 ± 107.0 [75.6; 623.0]	160.5 ± 119.7 [13.0; 783.6]	408.3 ± 160.3 [116.6; 1,050.8]	563.8 ± 202.5 [231.9; 1,146.2]	299.4 ± 190.8 [20.9; 1,266.4]

Abbreviation: DBH, diameter at breast height.

Terrestrial laser scanning data acquisition was carried out with a Trimble TX5 3D phase‐shift laser scanner (Trimble Navigation Limited) operating at a 1,550 nm wavelength and measuring 976,000 points per second, delivering a hemispherical (300° vertical × 360° horizontal) point cloud with an angular resolution of 0.009° in both vertical and horizontal direction with a maximum range of 120 m (resulting a point distance approximately 6.3 mm at 10 m distance) and beam divergence of 0.011°. All three study sites were scanned between September and October 2018 using a multiscan approach to minimize occlusion. Eight scans were conducted at each sample plot with two scans on two sides of a plot center and six auxiliary scans closer to the plot borders (see Figure 1 in Saarinen et al., 2020). Artificial targets (i.e., white spheres with a diameter of 198 mm) were placed around each sample plot to be used as reference objects for registering the eight scans into a single, aligned coordinate system with a FARO SCENE software (version 2018). The registration resulted in a mean distance error of 2.9 ± 1.2 mm, mean horizontal error was 1.3 ± 0.4 mm, and mean vertical error was 2.3 ± 1.2 mm. LAStools software (Isenburg, 2019) was used to remove topography from the point clouds by applying a point cloud normalization workflow presented by Ritter et al. (2017).

## 3 METHODS

### 3.1 TLS point cloud classification into stem and nonstem

**Figure 3:**
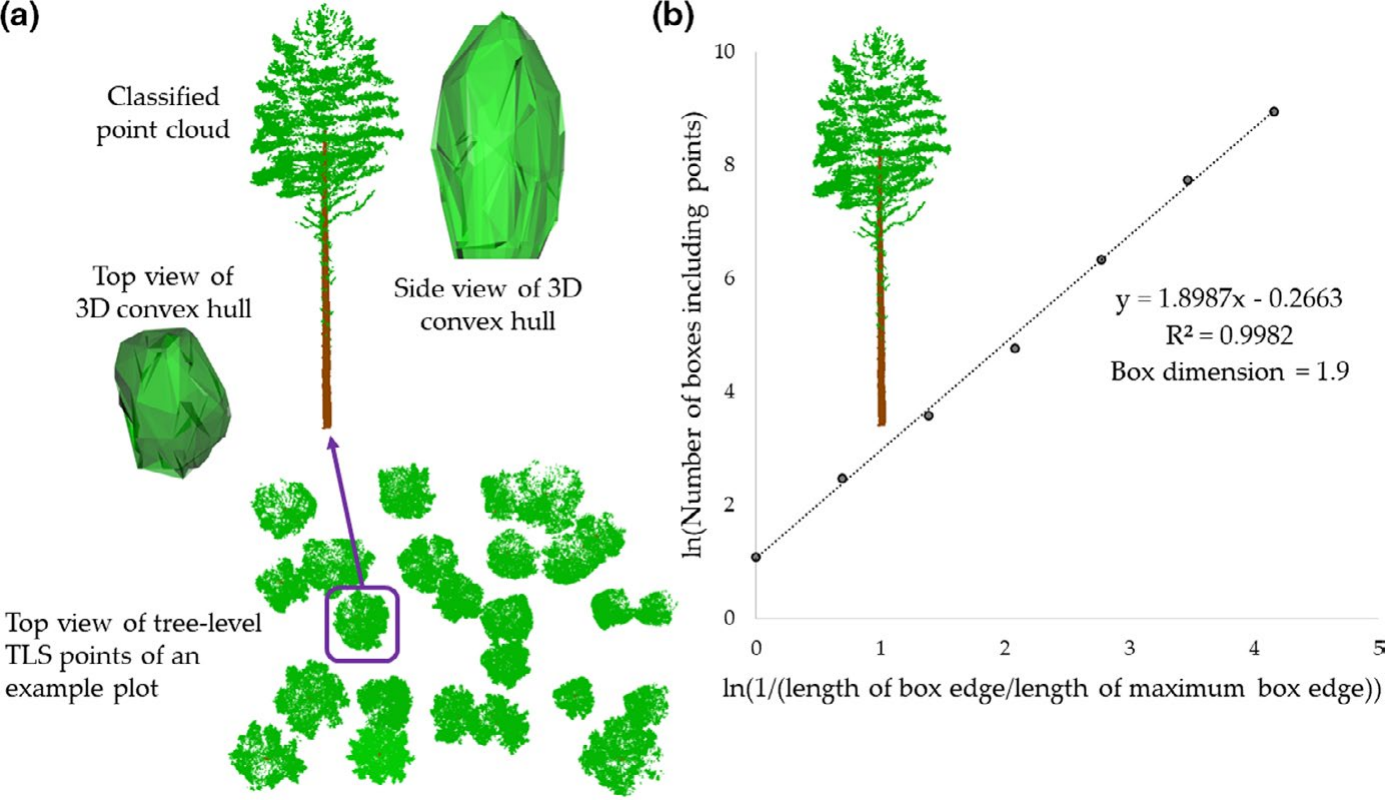
Crown‐segmented point clouds of individual Scots pine trees (a bottom) and an example of classified point clouds representing a Scots pine tree (a top center) with the fitted 3D convex hull enveloping the crown points, viewed from the top (a top left) and side (a top right). The definition for the box dimension for the same Scots pine (b), the slope of the fitted straight line (1.90) equals the box dimension, whereas the intercept (−0.27) is a measure of tree size and coefficient of determination (*R* ^2^ = 1.0) self‐similarity (Dorji et al., 2019). Modified after Figure 1 in Seidel (2018)

First, plot‐level TLS point clouds were segmented to identify points from individual trees. Local maxima from canopy height models (CHMs) with a 20‐cm resolution were identified using the Variable Window Filter approach (Popescu & Wynne, 2004), and the marker‐controlled watershed segmentation (Meyer & Beucher, 1990) was applied to delineate crown segments. A point‐in‐polygon approach was applied for identifying all points belonging to each crown segment. To identify points that originated from stem and crown within each crown segment, a point cloud classification procedure by Yrttimaa et al. (2020) was used. The classification of stem and nonstem points assumed that stem points have more planar, vertical, and cylindrical characteristics compared with nonstem points representing branches and foliage (Liang et al., 2012, Yrttimaa et al., 2020). The method by Yrttimaa et al. (2019, 2020) is an iterative procedure beginning from the base of a tree and proceeding toward treetop. More detailed description of the point cloud classification workflow can be found in Yrttimaa et al. (2019, 2020). The result of this step was 3D point clouds for each individual tree (*n* = 741) within the nine sample plots (Figure 3a).

### 3.2 Attributes for structural complexity, crown dimensions, benefit‐to‐cost ratio, growth, and light availability

Box dimension introduced by Seidel, Annighöfer, et al. (2019) was used for assessing structural complexity of the individual trees. Box dimension is a structural measure derived from individual tree TLS point clouds. First, one box including all TLS points of a single tree was fitted (i.e., initial box) in which the edge length of the box was tree height and then boxes of different sizes (i.e., tree height/2, tree height/4, tree height/8, tree height/16, tree height/32, tree height/64, tree height/128) were fitted to point clouds of each tree and the number of fitted boxes of each size was saved. Finally, the box dimension for each tree was defined as a slope between natural logarithm of 1/(box edge length of certain size/edge length of initial box) and natural logarithm of number of boxes including boxes of certain size (Figure 3b). Box dimension can theoretically vary between one and three, one representing pole‐like objects and three solid objects such as a cube.

Following examples by Seidel, Annighöfer, et al. (2019), the relationship between box dimension and attributes characterizing stem and crown size, and benefit‐to‐cost ratio, growth, and light availability were assessed. Stem attributes included DBH, tree height, and stem volume, whereas crown attributes included crown radius, crown projection area, and crown volume. Tree height was obtained using the height of the highest TLS point of each tree (i.e., normalized above ground), whereas DBH was defined from taper curve obtained with a combination of circle fitting to original stem points and fitting a cubic spline (see Saarinen et al., 2020; Yrttimaa et al., 2019). Stem volume, on the other hand, was defined by considering the stem as a sequence 10‐cm vertical cylinders and summing up the volumes of the cylinders using the estimated taper curve. Crown attributes were generated from TLS points originating from branches and foliage (i.e., crown points). A 2D convex hull was fitted to envelope the crown points of each tree of which crown projection area was derived, whereas crown volume was calculated from a 3D convex hull. Crown width, on the other hand, was defined as the distance between the two most outer points in xy space.

Benefit‐to‐cost ratio was defined as a ratio between crown surface area and stem volume (i.e., surface‐to‐volume ratio), which were used as proxies for the photosynthetically active surface and building costs of a tree, respectively. The crown surface area was calculated from a 3D convex hull fitted to crown points of each tree (Figure 3a). As TLS data were only acquired once from the study sites, growth of DBH, tree height, stem volume, and ΔH/DBH was calculated using field inventory measurements conducted in 2005–2006 and 2018–2019 for all live trees that were in the sample plots during the last field measurements. Light availability of tree crowns was assumed to be related to the level of competition each tree is facing, and the Hegyi index was used as a measure for competition and, thus, a proxy for the light availability (Seidel, Annighöfer, et al., 2019). Hegyi's competition index was calculated for each tree as follows:

(1) $${\text{Hegyi}}^{\prime }s\;\text{competition}\;\text{index}=\sum_{j=1}^{n}\frac{\frac{{\text{DBH}}_{i}}{{\text{DBH}}_{j}}}{{\text{dist}}_{\mathrm{ij}}},$$

where *i* is the subject tree for which competition index is calculated, *j* is a competitor, ${\text{dist}}_{\mathrm{ij}}$ is the distance between the subject tree *i* and the competitor *j*, and *n* is the number of competitors within 5 m radius around the subject tree *i*. The TLS‐based DBH of subject and competitor trees was used in calculating Hegyi's competition index, and the RMSE and bias are 0.7 cm (3.4%) and −0.1 (−0.6%), respectively (Yrttimaa et al., 2020).

### 3.3 Statistical analyses

Due to the data structure (i.e., several sample plots in each study site), a nested two‐level linear mixed‐effects model (Equation 2) was fitted using restricted maximum likelihood included in package nlme (Pinheiro et al., 2020) of the R‐software to assess the effects of thinning treatment on box dimension.

(2) $$\begin{array}{cc} y_{\mathrm{ij}} & =\beta_{1}Moderate\;below_{i}+\beta_{2}Moderate\;above_{i}+\beta_{3}Moderate\;systematic_{i}\\ & \;+\beta_{4}Intensive\;below_{i}+\beta_{5}Intensive\;above_{i}+\beta_{6}Intensive\;systematic_{i}\\ & \;+\beta_{7}No\;treatment_{i}+a_{i}+c_{\mathrm{ij}}+\epsilon_{\mathrm{ij}}, \end{array}$$

where $y_{\mathrm{ij}}$ is box dimension, $\beta_{1},\ldots \beta_{7}$ are fixed parameters; *i*, *i* = 1, …, *M*, refers to study site; *j*, *j* = 1, …, $n_{i}$, to a plot; and $a_{i}$ and $c_{\mathrm{ij}}$ are normally distributed random effects for sample plot *j* and for sample plot *j* within study site *i*, respectively, with mean zero and unknown, unrestricted variance–covariance matrix, and $\epsilon_{\mathrm{ij}}$ is a residual error with a mean zero and unknown variance. The random effects are independent across study sites, and sample plots and residual errors are independent across trees. The effects of a study site and a sample plot within the study sites on box dimension, crown and stem attributes, surface‐to‐crown ratio, growth, and light competition were assessed through their variances.

The analysis of variance utilizing the results from the nested two‐level linear mixed‐effects model was applied in testing the statistically significant difference in the box dimension affected by the thinning treatments, the study sites, and the plots within the study sites. Furthermore, to reveal the possible statistically significant difference in the box dimension between a thinning treatment against other treatments, Tukey's honest significance test was applied. To assess the relationship between structural complexity and stem and crown dimensions, benefit‐to‐cost ratio, and growth and light availability, similar approach was applied, but box dimension was added as a continuous predictor variable into Equation 2. Then, the response variable was a single stem and crown attribute, benefit‐to‐cost ratio, and growth attributes (i.e., DBH, height, stem volume, and ΔH/DBH) at a time.

The analysis of variance was applied to investigate the significance of the relationship between box dimension and thinning treatment, whereas Tukey's honest significance test was used for revealing difference in architectural attributes (stem and crown dimensions, benefit‐to‐cost ratio, growth, and light availability) between thinning intensity. Finally, Pearson's correlation coefficient and coefficient of determination (*R* ^2^) were calculated between box dimension and stem and crown attributes, and benefit‐to‐cost ratio, growth attributes, and competition index for each thinning treatment to assess their relationships.

## 4 RESULTS

**Figure 4:**
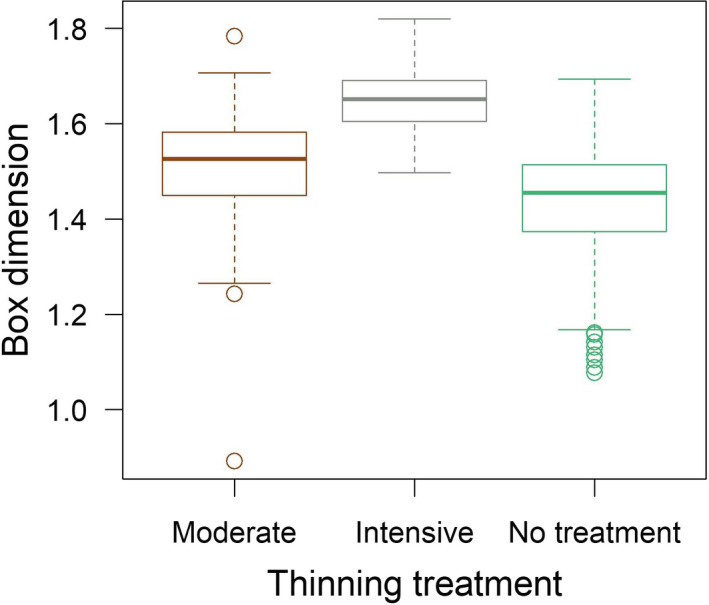
Variation in box dimension between moderate (i.e., state‐of‐the‐art thinning in Finland), intensive (i.e., 50% more basal area was removed compared to moderate) thinning, and without thinning (i.e., no treatment since establishment of the study sites)

**Figure 5:**
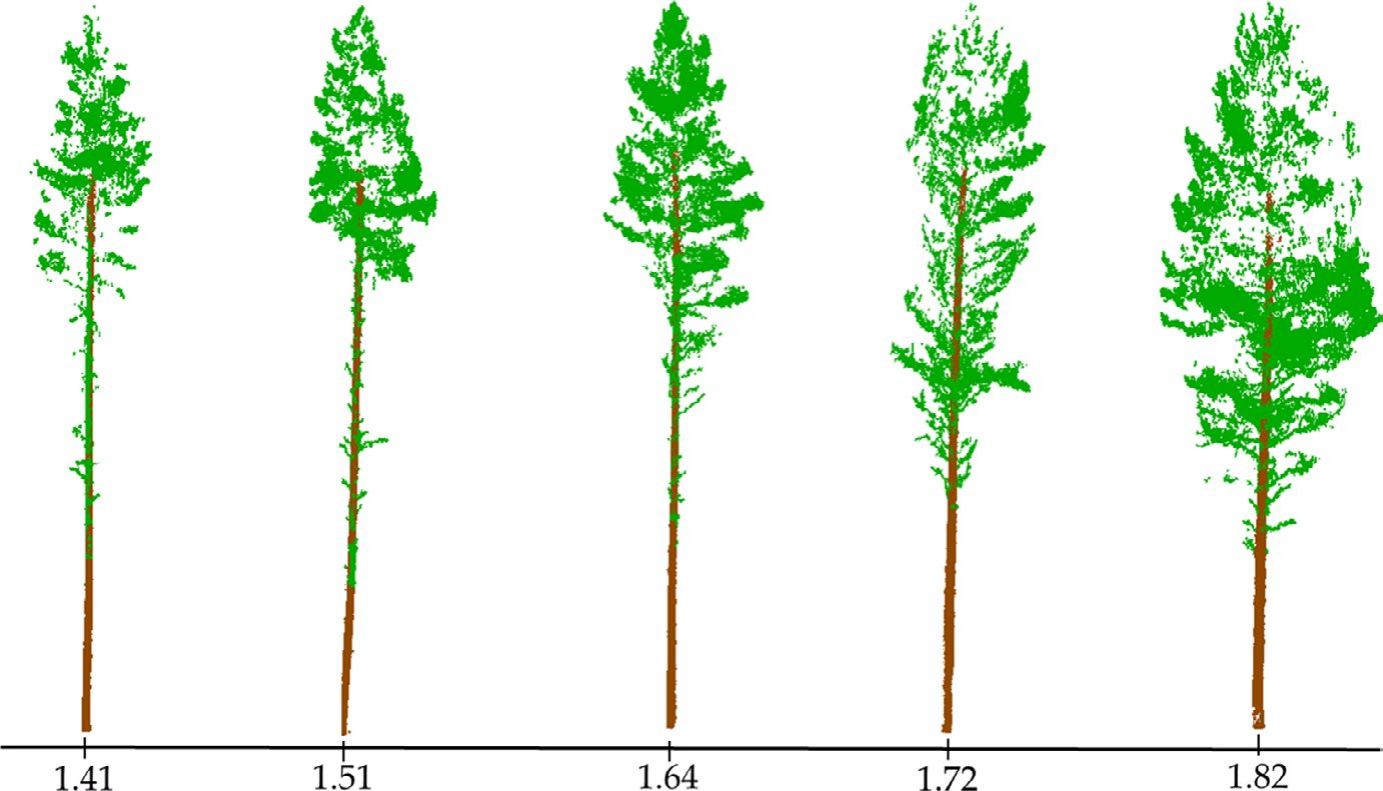
Terrestrial laser scanning point clouds from example trees with varying structural complexity (i.e., box dimension)

The box dimension was 1.5 ± 0.1 and 1.6 ± 0.1 for moderate and intensive thinnings, respectively, whereas for control plots it was 1.4 ± 0.1, indicating increasing structural complexity of individual trees as the thinning intensity increased. The linear mixed‐effects model and the analysis of variance revealed statistically significant difference (*p* < .01) in box dimension between thinning treatments (Figure 4). Trees with small differences in box dimension can look very different as depicted in Figure 5.

When assessing drivers of structural complexity, all stem, crown, and growth attributes were found significant (*p* < .05) in the nested two‐level linear mixed‐effects models (Table 3). Benefit‐to‐cost ratio and light availability, on the other hand, were not. Intensive thinning increased tree height, benefit‐to‐cost ratio, height growth, and light availability significantly (*p* < .05), whereas benefit‐to‐cost ratio, height growth, and light availability were significantly (*p* < .05) smaller in the linear mixed‐effects models (Table 3). However, coefficient of determination was <0.2 for all other architectural attributes (Figures S1–S4) except for crown dimensions where it was 0.5 between box dimension and crown projection area and crown volume (Figure S5).

**Table 3:** Results of the nested two‐level linear mixed‐effects models with box dimension as dependent variable and thinning treatment together with stem and crown attributes, benefit‐to‐cost ratio, growth attributes, and light availability as independent variables

Attribute	Model parameter	Estimate	*SE*	*p*‐Value
*Stem attributes*				
DBH (cm)	DBH	0.001	0.001	.000*
	Moderate	1.312	0.031	.000*
	Intensive	0.087	0.036	.074
	No treatment	−0.055	0.035	.191
Height (m)	Height	0.001	0.002	.000*
	Moderate	1.369	0.043	.000*
	Intensive	0.124	0.035	.025*
	No treatment	−0.082	0.034	.074
Stem volume (dm^3^)	Stem volume	0.000	0.000	.000*
	Moderate	1.443	0.028	.000*
	Intensive	0.096	0.038	.064
	No treatment	−0.066	0.037	.148
*Crown attributes*				
Crown width (m)	Crown width	0.065	0.004	.000*
	Moderate	1.232	0.025	.000*
	Intensive	0.064	0.027	.078
	No treatment	−0.032	0.026	.282
Crown projection area (CPA) (m^2^)	CPA	0.014	0.001	.000*
	Moderate	1.356	0.022	.000*
	Intensive	0.023	0.024	.395
	No treatment	−0.018	0.022	.469
Crown volume (m^3^)	Crown volume	0.001	0.000	.000*
	Moderate	1.364	0.027	.000*
	Intensive	0.038	0.301	.282
	No treatment	−0.025	0.030	.458
*Benefit‐to‐cost ratio*				
Surface‐to‐volume ratio (StV)	StV	0.000	0.000	.063
	Moderate	1.154	0.022	.000*
	Intensive	0.125	0.031	.015*
	No treatment	−0.092	0.029	.036*
*Growth attributes*				
DBH growth (cm)	DBH growth	0.023	0.003	.000*
	Moderate	1.149	0.025	.000*
	Intensive	0.065	0.033	.118
	No treatment	−0.041	0.003	.259
Height growth (m)	Height growth	0.016	0.004	.000*
	Moderate	1.442	0.031	.000*
	Intensive	0.131	0.029	.011*
	No treatment	−0.080	0.028	.046*
Volume growth (dm^3^)	Volume growth	0.000	0.000	.000*
	Moderate	1.452	0.024	.000*
	Intensive	0.085	0.033	.062
	No treatment	−0.056	0.032	.155
ΔH/DBH	ΔH/DBH	0.526	0.081	.000*
	Moderate	1.025	0.071	.000*
	Intensive	0.065	0.033	.125
	No treatment	−0.041	0.032	.271
*Light availability*				
Competition index (CI)	CI	0.004	0.004	.279
	Moderate	1.518	0.022	.000*
	Intensive	0.120	0.033	.022*
	No treatment	−0.095	0.031	.040*

* denotes statistical significance of *p*‐value < .05.

Thinning treatment was statistically significant (*p* < .05) in mixed‐effects models where height and volume growth, benefit‐to‐cost ratio, light availability, and each stem attribute were included as predictor variable at a time. Tukey's honest significance test revealed that there was a statistical difference (*p* < .05) between moderate and intensive thinning in all stem attributes, benefit‐to‐cost ratio, height and volume growth, and light availability. Thinning, either moderate or intensive, resulted in significant difference (*p* < .05) in all stem attributes, crown width, benefit‐to‐cost ratio, all growth attributes, and light availability when compared to trees without a thinning treatment.

Pearson's correlation coefficient was > 0.5 between box dimension and crown projection area and crown volume with moderate and no thinning treatments (Table 4). However, the correlation was significant (*p* < .001) between box dimension and all crow attributes with all thinning treatments. However, the correlation between box dimension and stem attributes, benefit‐to‐cost ratio, growth, and light availability was mostly < 0.5 indicating a weak relationship between them. Nevertheless, Pearson's correlation coefficients were found significant for stem attributes in moderate thinning and for growth attributes in control plots.

**Table 4:** Pearson's correlation coefficients between box dimension and stem and crown attributes, benefit‐to‐cost ratio, growth attributes, and light availability grouped by thinning treatment

	Moderate	Intensive	No treatment
	Box dimension		
*Stem attributes*			
DBH (cm)	0.32*	0.27	0.33*
Height (m)	0.28*	−0.08	−0.01
Stem volume (dm^3^)	0.24*	0.17	0.24
*Crown attributes*			
Crown width (m)	0.55*	0.36*	0.56*
Crown projection area (m^2^)	0.69*	0.43*	0.62*
Crown volume (m^3^)	0.67*	0.37*	0.57*
*Benefit‐to‐cost ratio*			
Surface‐to‐volume ratio	0.37*	0.00	0.06
*Growth attributes*			
DBH growth (cm)	0.09	0.34*	0.43*
Height growth (m)	−0.03	−0.09	0.23*
Volume growth (dm^3^)	0.26*	0.23	0.25*
ΔH/DBH	0.07	0.34*	0.33*
*Light availability*			
Competition index	−0.25*	0.06	0.08

Note

Correlation coefficients > 0.50 are bolded, and * denotes statistical significance (*p* < .001).

## 5 DISCUSSION

Thinning increased the structural complexity of individual Scots pine trees confirming our hypothesis H1. Crown projection area showed a positive relationship (mean correlation coefficient > 0.6) with structural complexity, whereas tree height did not, leading to partially accepting the hypothesis about associations between structural complexity and horizontal and vertical measures of Scots pine trees (H2). Finally, the hypothesis H3 is also partly accepted as there was practically no relationship between structural complexity and benefit‐to‐cost ratio, tree growth, or the light availability, but crown and growth attributes were, however, significant when estimating structural complexity. Forest management, and thinning especially, affected structural complexity of individual Scots pine trees. Stem, crown, and growth variables were found significant predictors for it indicating them as drivers for structural complexity.

Crown dimensions affected structural complexity more than stem attributes (i.e., DBH, tree height, and volume) which is similar to the findings of Seidel, Ehbrecht, et al. (2019) who studied the relationship between structural complexity and stem and crown attributes of four deciduous species (i.e., *Fagus sylvatica* L., *Fraxinus excelsior* L., *Acer pseudoplatanus* L., and *Carpinus betulus* L.). Thinning affected stem or growth attributes, benefit‐to‐cost ratio, and light availability of Scots pine trees, as either moderate or intensive thinning resulted in differing values for these attributes compared with Scots pine trees without a thinning treatment (i.e., control plots).

There was no relationship (*R* ^2^ < 0.2 and correlation coefficient < 0.1, not significant) between structural complexity and benefit‐to‐cost ratio indicating that it does not affect structural complexity of Scots pine trees. This is contradictory to the findings by Seidel, Annighöfer, et al. (2019) who found *R* ^2^ of at least 0.25 between structural complexity and cost‐to‐benefit ratio for 76 deciduous trees (i.e., 46 *Fagus sylvatica* L., 25 *Fraxinus excelsior* L., and 5 *Acer pseudoplatanus* L.). Their study site was a mixed, unmanaged deciduous forest and there were ~150 trees/ha, whereas in our study the tree density per ha was at least twice of that. Thus, it can be assumed that there was more space for the deciduous trees to grow. Additionally, tree form in general is different between conifers and deciduous trees due to the difference in their shoot growth, in other words the degree of apical dominance differs being strong for conifers and weaker for deciduous trees (Kozlowski, 1964). This can produce differences in structural complexity and its relationship to benefit‐to‐cost ratio.

Light availability (measured through competition) did not affect structural complexity of Scots pine trees, and there was no relationship between them. However, larger variation in light availability was found in plots without thinning treatments, which is expected as it has shown in previous studies that thinning decreases competition (Jacobs et al., 2020; Juchheim et al., 2017; Mäkinen & Isomäki, 2004). Seidel, Annighöfer, et al. (2019) reported decreasing structural complexity when competition increased, but their study only included 93 red oak (*Quercus rubra* L.) trees from 10 different sites compared with our ~ 740 Scots pine trees. Furthermore, Dorji et al. (2019) showed that competition reduced structural complexity of European beech trees. Similar to the results of benefit‐to‐cost ratio discussed above, deciduous trees have a different branching pattern compared with conifers. Future research should focus on the response of structural complexity to light availability of different species with enough trees to clearly understand their relationship.

Ehbrecht et al. (2017) studied stand structural complexity in mixed stands with even‐aged and uneven‐aged forest management. They discovered that stand structural complexity differed between tree species and even‐aged coniferous stands had less complex structure compared with *Fagus sylvatica* (L.) stands. We only studied one tree species, namely Scots pine, but found that structural complexity varied between trees with different management history, although all the plots were even‐aged and single layer that could have decreased their structural complexity.

Forest structural complexity has been widely studied (Camarretta et al., 2020; Ishii et al., 2004; McElhinny et al., 2005), but there is less research on tree structural complexity, which was the focus of this study. TLS can provide traditional tree attributes (i.e., DBH, volume, crown dimensions) for characterizing tree structure, but its potential can also be expanded for new attributes. Here, we only utilized box dimension to measure structural complexity, which can be considered as a weakness of the study as we cannot compare these results to other measures. However, the box dimension was chosen because it was easy to calculate, it has shown its potential in characterizing individual tree structure (Seidel, Annighöfer, et al., 2019), and because no other measures for individual tree structural complexity were found in the literature. As it was shown, there was a relationship between box dimension and stem, crown, and growth attributes indicating that these attributes can explain structural complexity if individual trees. None of them, however, considers the entire 3D tree structure to which the box dimension brings added value. The strength of the study is that the study design allowed us to compare forest management practices without confounding factors that could have been a challenge in uneven‐aged or mixed‐species stands.

Forest management in general, and thinning in particular, controls the between‐tree competition especially by removing part of shadowing canopy mass to enhance the growth of remaining trees (White, 1980). Although there is a strong economic incentive in forest management for wood production (Puettmann et al., 2015), there is an increasing understanding how forest management can also be applied for supporting diversity of forest structure (Bergeron et al., 2002; Kuuluvainen, 2009), biodiversity (Fedrowitz et al., 2014), resilience (Messier et al., 2013), and carbon uptake (Hardiman et al., 2011). Additionally, there is an increasing respect toward recreational opportunities and landscape amenities (Butler & Leatherberry, 2004; Hugosson & Ingermason, 2004; Urquhart & Cortney, 2011). Therefore, structural diversity has been identified as a silvicultural principle that could be addressed with nonconventional forest management practices (Puettmann et al., 2015). Furthermore, forest structural variety can be used as a measure for biodiversity and structurally complex forests enhance carbon uptake (Gough et al., 2019), which are important ecosystem services provided by forests. Thus, it can be expected that structural diversity can also be beneficial for functional and species diversity of forests.

## 6 CONCLUSIONS

Structural diversity at different scales can be linked to biodiversity and carbon uptake, as well as attractiveness of landscapes and recreational activities. Possibilities for forest management in considering these more varied objectives are acknowledged, but objective measures of structural diversity have been lacking as 3D information on structure of forests and trees has practically been unavailable before various laser scanning sensors. This study provides an example how structural complexity of individual trees can be quantitatively assessed and how it is affected by forest management. We demonstrated the use of so‐called box dimension in characterizing structural complexity of individual Scots pine trees in managed plots. Thinning intensity affected structural complexity, and intensive thinning resulted in increased structural complexity. Increasing crown size (i.e., width, surface area, and volume) and tree growth also increased structural complexity, but there was no relationship between structural complexity and benefit‐to‐cost ratio (i.e., relationship between crown surface area and stem volume) or light availability (i.e., competition). More research is required to better understand the specific drivers behind structural complexity of different tree species. For example, how regeneration and growth of other than traditional attributes (e.g., DBH and height) affect the structural complexity. It can be concluded that thinning had an effect on structural complexity of individual Scots pine trees and stem, crown, and growth attributes were identified as drivers for the structural complexity in managed boreal forests.

## CONFLICT OF INTEREST

None declared.

## AUTHOR CONTRIBUTION

**Ninni Saarinen:** Conceptualization (lead); Data curation (lead); Formal analysis (lead); Funding acquisition (lead); Investigation (lead); Methodology (equal); Project administration (lead); Supervision (lead); Validation (lead); Visualization (lead); Writing‐original draft (lead); Writing‐review & editing (lead). **Kim Calders:** Conceptualization (equal); Funding acquisition (equal); Writing‐original draft (equal); Writing‐review & editing (equal). **Ville Kankare:** Data curation (equal); Software (equal); Writing‐original draft (equal); Writing‐review & editing (equal). **Tuomas Yrttimaa:** Data curation (equal); Software (equal); Writing‐original draft (equal); Writing‐review & editing (equal). **Samuli Junttila:** Funding acquisition (supporting); Writing‐original draft (equal); Writing‐review & editing (equal). **Ville Luoma:** Data curation (equal); Investigation (supporting); Writing‐original draft (equal); Writing‐review & editing (equal). **Saija Huuskonen:** Resources (equal); Writing‐original draft (equal); Writing‐review & editing (equal). **Jari Hynynen:** Resources (equal); Writing‐original draft (equal); Writing‐review & editing (equal). **Hans Verbeeck:** Conceptualization (supporting); Writing‐original draft (equal); Writing‐review & editing (equal).

## Supporting information

Figures S1–S5

## Data Availability

Data used for calculating box dimension are available for download on Zenodo (http://doi.org/10.5281/zenodo.4419878).
