# Supplementary material for: Understanding 3D structural complexity of individual Scots pine trees with different management history
Source: Ecol Evol. 2021 Jan 31;11(6):2561–72. doi: 10.1002/ece3.7216 (PMC7981231; doi:10.1002/ece3.7216)
Supplement: Supplementary file 1 — Figures S1–S5 [file ECE3-11-2561-s001.docx]

**Supplementary material**


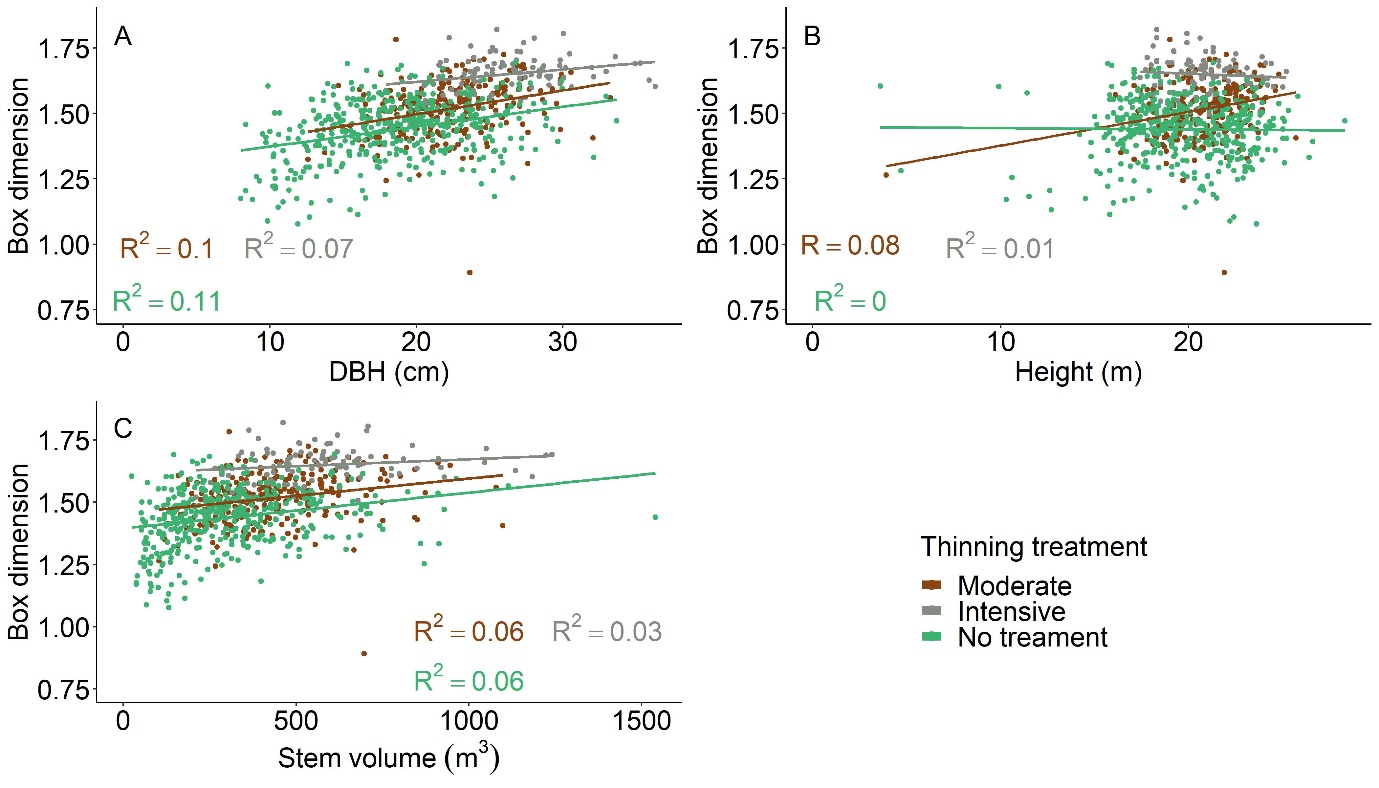
Figure S1. Relationship between box dimension and diameter at breast height (DBH) (A), tree height (B), and stem volume (C) grouped by thinning treatment.


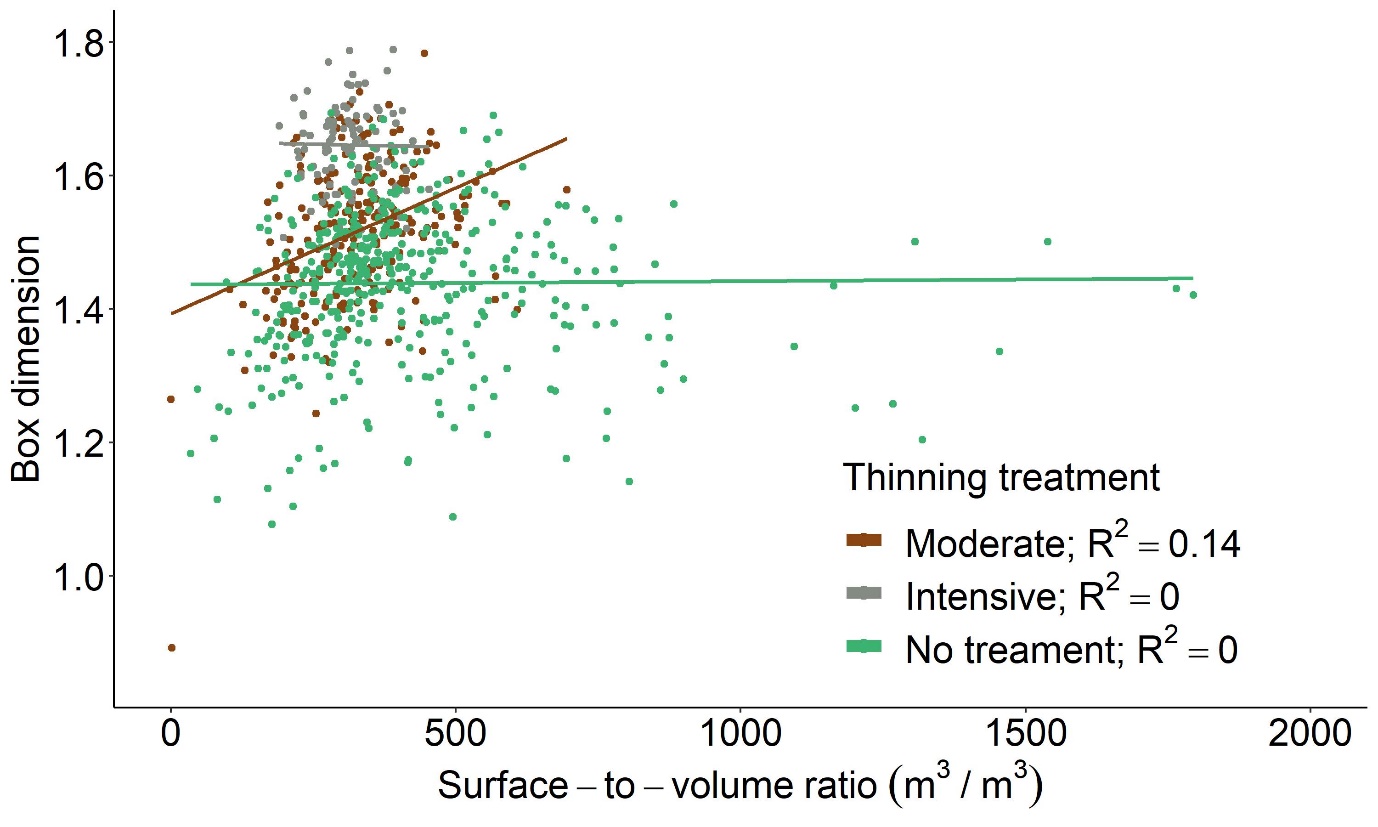
Figure S2. Regression lines and scatterplots of box dimension against surface-to-volume ratio (a proxy for a relationship between photosynthetically active surface and building costs of a tree) of Scots pine trees grouped by thinning treatment.


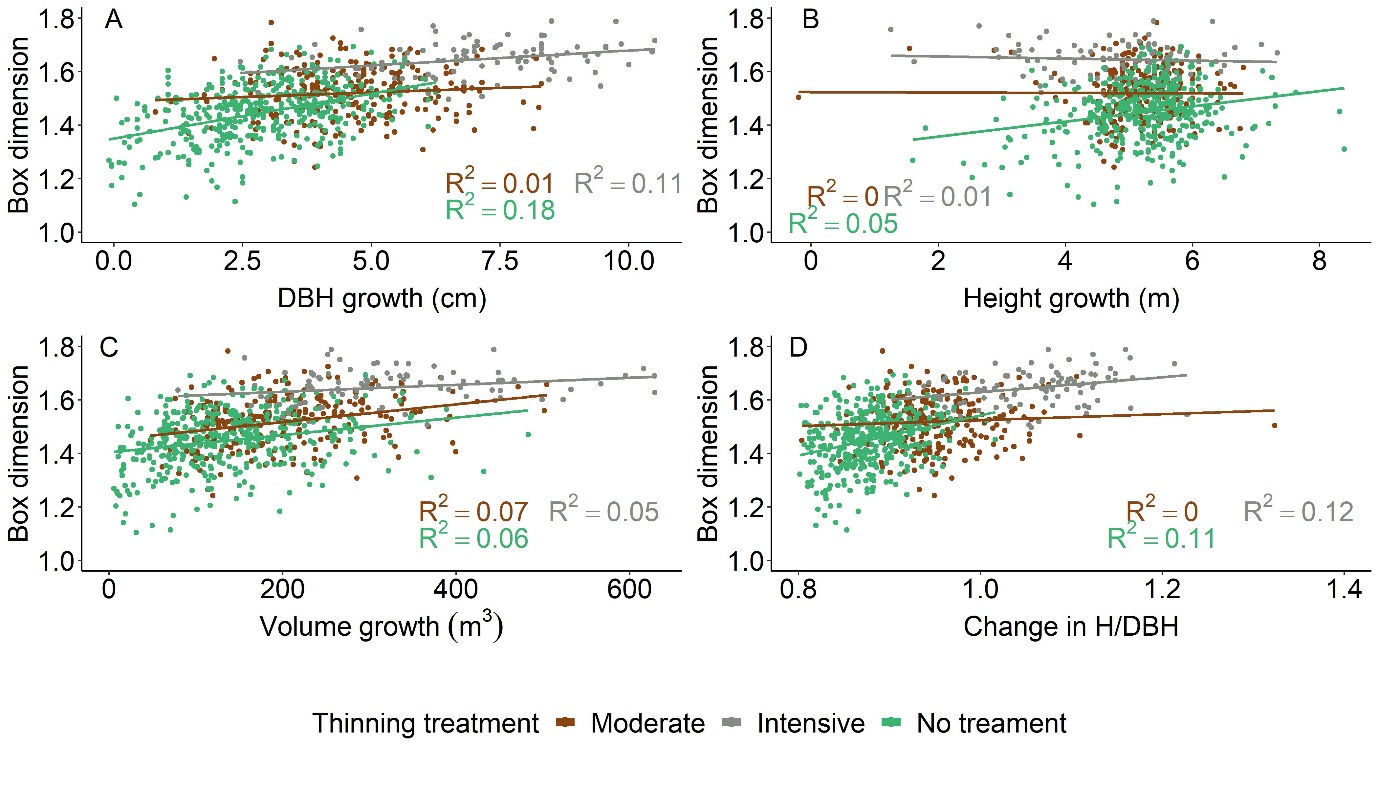
Figure S3. Relationship between box dimension and growth of diameter at breast height (DBH) (A), height (B) and stem volume (C) as well as change in height-DBH ratio (ΔH/DBH) (D) grouped by thinning treatment.


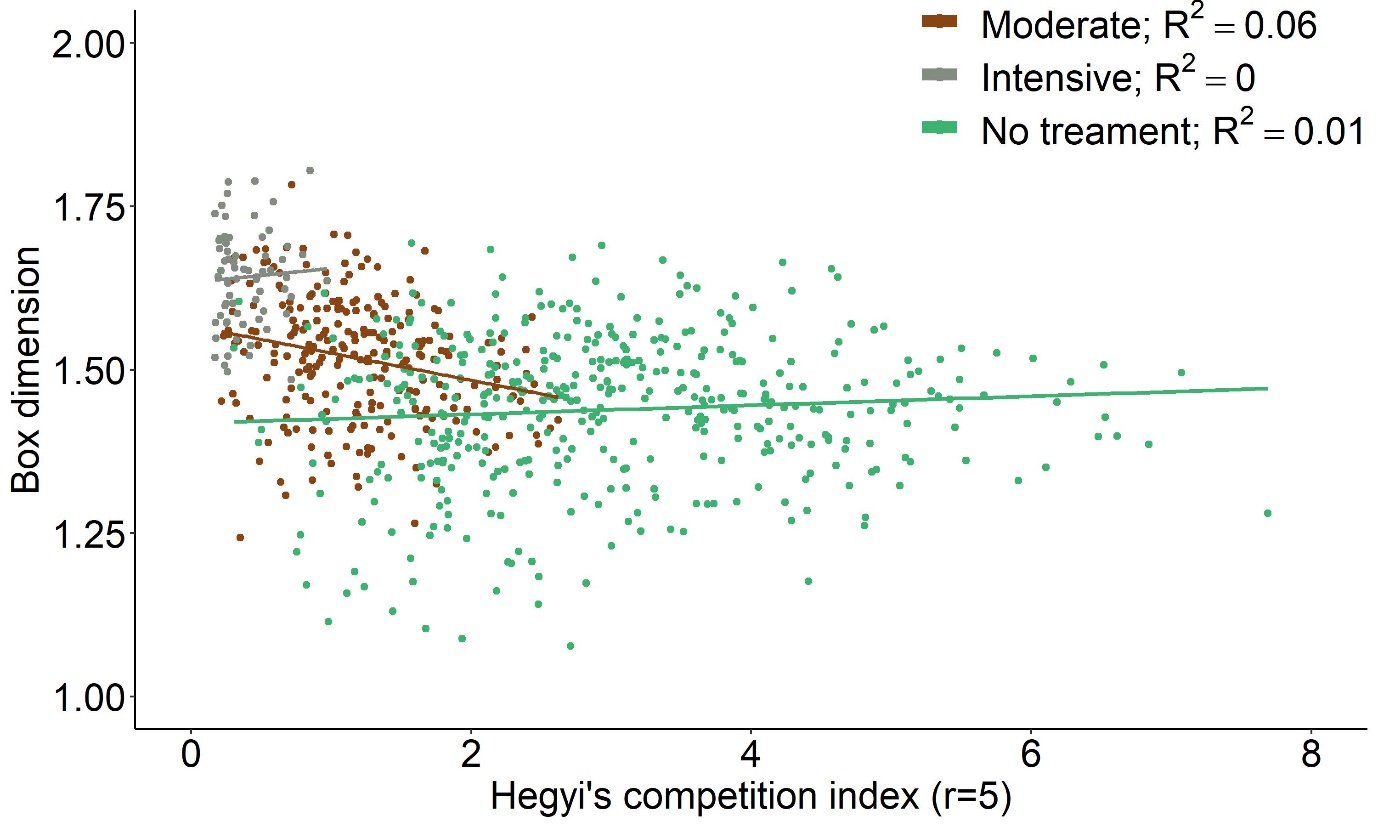
Figure S4. Regression lines and scatterplots of Hegyi’s competition index against box dimension of Scots pine trees grouped by thinning treatment.


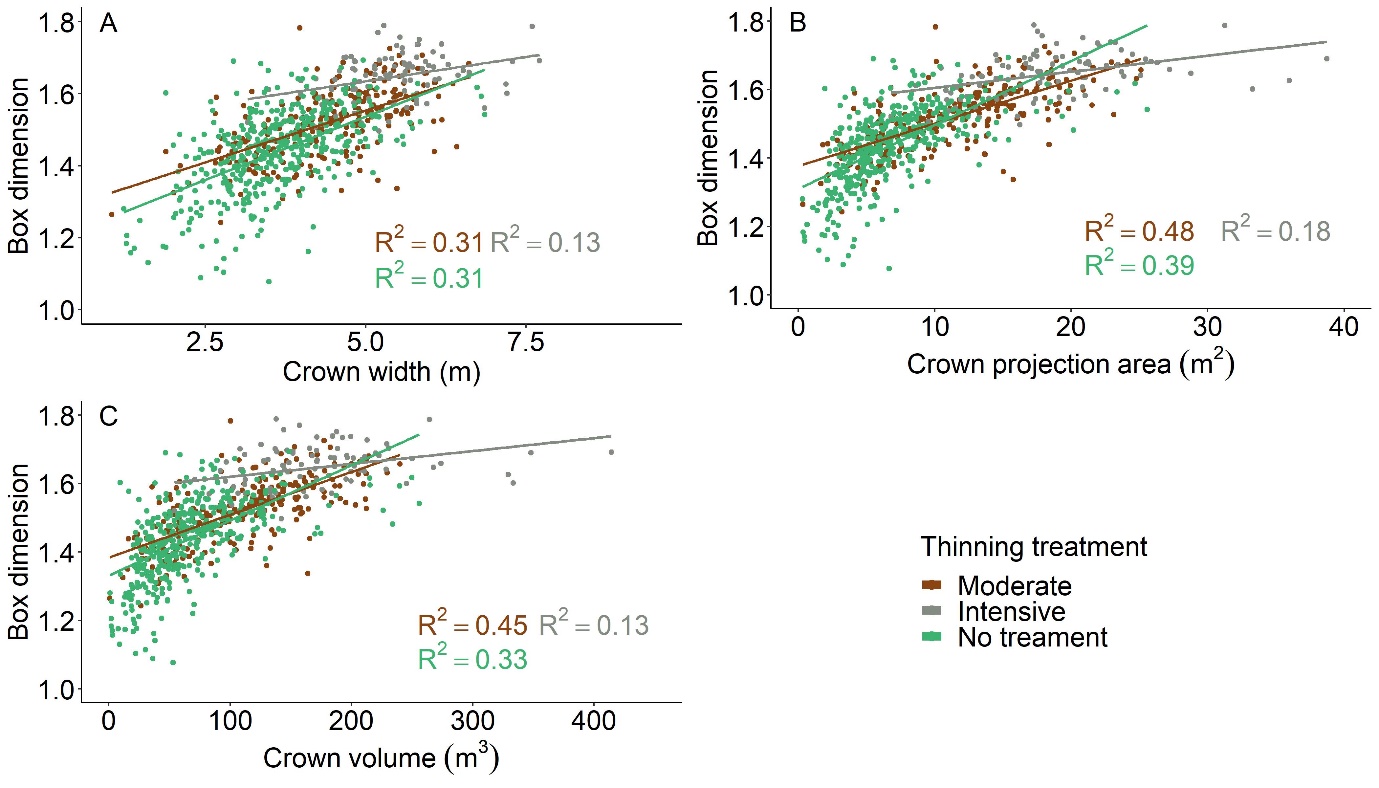
Figure S5. Relationship between box dimension and crown width (A), crown projection area (B), and crown volume (C) grouped by thinning treatment.
